# Feeding management before gastrointestinal studies in pigs

**DOI:** 10.1177/0023677220960509

**Published:** 2020-10-12

**Authors:** Rachael Gregson, Stephen Greenhalgh, Benjamin Cox, Sandy Cochrane, R Eddie Clutton

**Affiliations:** 1 Wellcome Critical Care Laboratory for Large Animals, Dryden Farm, University of Edinburgh, UK; 2 Division of Imaging and Technology, School of Medicine, University of Dundee, UK; 3 James Watt South Building, James Watt School of Engineering, University of Glasgow, UK

**Keywords:** Pigs, anaesthesia, food withdrawal, refinement, gastrointestinal tract, capsule endoscopy, colorectal surgery

## Abstract

Pigs are used to model humans in gastrointestinal (GI) studies because of their comparable size, physiology and behaviour: both are monogastric omnivores. A porcine surgical model for testing novel, tethered ultrasound capsule endoscopes (USCE) requires a clean, motile small intestine. Recommendations for human GI tract preparation before the mechanically similar process of video capsule endoscopy describe using oral purgatives, while high-carbohydrate drinks are recommended before colorectal surgery. Reports of the GI preparation of pigs exist but lack technical details, that is, administration, efficacy and side effects. This report details feeding a high-energy liquid diet to 11 female pigs undergoing surgery and USCE which was readily accepted and easily administered, and which produced a clean, motile small intestine and caused no detectable physiological/behavioural abnormalities.

## Résumé

Les porcs sont utilisés pour modéliser les humains dans les études gastro-intestinales en raison de leur taille, de leur physiologie et de leur comportement comparables: les deux sont des omnivores monogastriques. Un modèle chirurgical porcin pour tester de nouveaux endoscopes par capsule ultrasonore à attache (USCE) nécessite un intestin grêle propre et motile. Les recommandations pour la préparation du tractus gastro-intestinal humain avant le processus mécaniquement similaire d'endoscopie vidéo par capsule décrivent l’utilisation de purgatifs oraux alors que les boissons à haute teneur en glucides sont recommandées avant une chirurgie colorectale. Il existe des rapports sur la préparation gastro-intestinale des porcs, mais sans aucun détails techniques concernant l’administration, l’efficacité, les effets secondaires. Ce rapport détaille l’alimentation liquide très énergétique de onze porcs femelles subissant une intervention chirurgicale et une USCE qui a été facilement acceptée, facilement administrée, a permis d’obtenir un petit intestin propre et motile et n’a causé aucune anomalie physiologique ou comportementale détectable.

## Abstract (de)

Schweine werden aufgrund ihrer vergleichbaren Größe, Physiologie und ihres Verhaltens als Modell für Menschen in gastrointestinalen Studien verwendet. Beide sind monogastrische Allesfresser. Für ein chirurgisches Modell vom Schwein zum Testen neuartiger, angebundener Ultraschall-Kapselendoskope (USCE) war es erforderlich, einen sauberen, beweglichen Dünndarm zu gewährleisten. Empfehlungen für die Vorbereitung des menschlichen Gastrointestinaltraktes vor dem mechanisch ähnlichen Verfahren der Videokapselendoskopie beschreiben die Verwendung oraler Abführmittel, während kohlenhydratreiche Getränke vor einer kolorektalen Operation empfohlen werden. Es gibt Berichte über die Magen-Darm-Präparation von Schweinen, aber es fehlen technische Details, z. B. zu Verabreichung, Wirksamkeit, Nebenwirkungen. Dieser Bericht beschreibt die Fütterung von elf weiblichen Schweinen, die einer Operation und USCE unterzogen wurden, mit einem hochenergetischen Flüssigfutter, das bereitwillig akzeptiert wurde, leicht zu verabreichen war, einen sauberen, beweglichen Dünndarm bewirkte und keine erkennbaren physiologischen/Verhaltensauffälligkeiten verursachte.

## Resumen

Los cerdos son utilizados en estudios gastrointestinales enfocados a humanos debido a que tienen un tamaño, una fisiología y un comportamiento parecido al de los humanos: ambos son omnívoros monogástricos. Un modelo quirúrgico porcino para probar unas nuevas cápsulas endoscópicas con tecnología de ultrasonido (USCE) y sujeción requería un intestino delgado de alta calidad y limpio. Se recomienda utilizar purgantes orales para la preparación del tracto gastrointestinal humano antes de realizar un proceso con videocápsula de endoscopia mecánicamente similar, mientras que para la cirugía colorrectal se recomiendan bebidas altas en carbohidratos. Existen informes de preparación gastrointestinal de cerdos pero hay una carencia de datos técnicos, como la administración, la eficacia y los efectos secundarios. Este estudio describe la administración de una dieta con líquidos de alta energía a once cerdas antes de someterse a una intervención quirúrgica y USCE; dicha dieta fue aceptada inmediatamente, se administró con facilidad, generó un intestino delgado de alta calidad y limpio, y no produjo ninguna anormalidad fisiológica/de comportamiento detectable.

## Introduction

Preparatory measures before gastrointestinal (GI) endoscopy aim to provide a view of the intestinal mucosa unobscured by turbid liquid or food material, both of which reduce diagnostic value.^1^ Similar measures reduce post-surgical complications such as wound dehiscence or anastomotic leakage.^2^ Pigs and humans are both monogastric omnivores, and similar pre-endoscopic preparation should be required. However, opinions regarding pre-procedural preparation for video capsule endoscopy (VCE) remain divided.

In humans, overnight provision of a liquid diet does not worsen small intestinal conditions compared to oral purgatives (sodium picosulphate/magnesium sulphate or polyethylene glycol)^1^ which are used for mechanical bowel preparation to empty the GI tract of faeces. However, oral purgative administration is not routine before VCE. Preparation should be guided by patient/clinical requirements,^3^ and consideration of pre-existing co-morbidities and perioperative antibiosis are considered more important in avoiding complications.^2^

Comparable preoperative preparation for laboratory pigs is sparsely described and lacks technical details.^4^ Complan® (liquid meal replacer) has been used to prepare pigs’ GI tract before endoscopic surgery,^5^ and a combination of an ‘electrolyte-rich liquid’ and mechanical bowel preparation has been used before anastomotic surgery in minipigs.^6^ Both methods were used for 48 hours before surgery without complications/results reported.

A clean, empty yet motile bowel was desired in terminally anaesthetised pigs in studies involving stomata formation and ultrasound capsule endoscopy (USCE)^7^ prototype testing. Here, the development of a method using a high-energy liquid diet to prepare commercial pigs is detailed.

## Methods

Following ethical approval by Roslin Institute's Animal Welfare Ethical Review Body, studies were conducted under Procedure Project Licence PF5151DAF. Eleven female commercial hybrid pigs, with a median body mass of 47 kg (range 35–50 kg) and a median age of 14 weeks (range 11–14 weeks), were delivered within 7 days of the start of the study. Pigs were pair-housed without straw or ingestible bedding. Rubber matting and heat lamps were used to maintain environmental conditions, which were enriched with dog toys and traffic cones.

**Figure 1.**
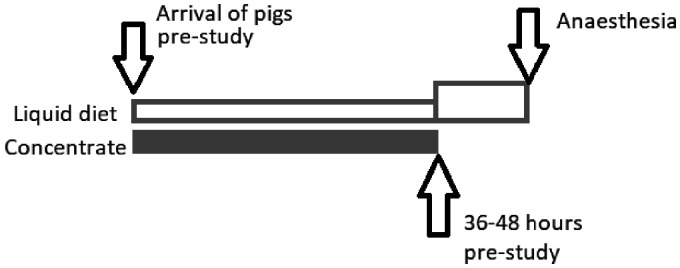
Feeding plan for pigs prior to gastrointestinal surgery and capulsar endoscopy.

A commercially available ‘dietetic feed source’ (Glutalyte®; Norbrook, Newry, UK) for use in calves with digestive disturbances was the chosen liquid diet. Prepared according to the manufacturer’s recommendations, it was provided in shallow troughs from arrival so that accustomisation could occur. Initially, 2 L was offered to each pen (two pigs) every 12 hours. Concentrated feed (ABN Pig Rearer Pellets; ABN Feeds, Cupar, UK) was offered twice daily until 36–48 hours before anaesthetic induction. After concentrate feeding stopped, Glutalyte® was offered at an increased rate (4 L/pen every 12 hours) until pre-anaesthetic medication was administered (Figure 1); water was always available *ab libitum*.

Intramuscular sedation comprising midazolam (0.25 mg/kg; Hypnovel; Roche, Welwyn Garden City, UK), morphine (0.25 mg/kg; morphine sulphate; Martindale Pharma, Brentwood, UK), medetomidine (7 µg/kg; Medetor; Dechra, Shrewsbury, UK) and ketamine (7 mg/kg; Ketamidor; Chanelle Pharma, Loughrea, Ireland) preceded induction/maintenance of anaesthesia with isoflurane (IsoFlo; Abbot, Maidenhead, UK) vaporised in medical air/oxygen. Blood glucose (BG) was monitored intermittently during anaesthesia (standard institution practice). After surgery, pigs were euthanised using pentobarbital (Pentoject 20%; Animalcare, York, UK) without recovery from anaesthesia. Descriptive statistics are stated as median (range).

## Results

Anaesthesia duration was five hours (4–11 hours). Glucose supplementation was required in 1/11 animals when BG was 2.4 mmol/L during surgery (normal >4.7 mmol/L),^8^ but this then normalised after intravenous supplementation (60–300 mg/kg/h; Glucose Intravenous Infusion 50% w/v; Hameln Pharma, Gloucester, UK). The small intestinal lumen was consistently empty of ingesta, and peristaltic motion was observed during surgery. No pigs showed abnormal behaviours prior to anaesthesia. All studies were completed successfully.

## Discussion

Providing a liquid diet in preparation for GI surgery helped maintain normal physiology, avoided oral purgatives and caused no observable undesirable effects on the pigs’ behaviour.

Initially, replacement of ingestible bedding with rubber mats in 2 m^2^ pens caused problems with soiling, as pigs lay in faeces-contaminated areas. Doubling the pen size and elevating sleeping areas allowed pigs to choose distinct sleeping and dunging areas, greatly improving cleanliness. Provision of robust manipulatable objects contributed to normal behaviour.

Since liquid or electrolyte-rich diets prepare the porcine GI tract adequately for surgery^5,6^ and a clear liquid diet provides suitable conditions for VCE in humans,^1^ it was decided to base GI preparation on a liquid diet. Mechanical bowel preparation using oral purgatives was avoided, as their usefulness is questionable^1,3^ and can cause adverse side effects in humans.^1^ Bowel preparation using prolonged food withdrawal was also undesirable because of adverse welfare effects. Glutalyte® was chosen because of its high carbohydrate (75.7% dextrose w/w) and glutamine content. Dextrose provides calories without fibre, avoiding accumulation of intraluminal contents, and glutamine is a ‘conditionally essential’ nutrient for enterocytes during periods of stress.^9^ Pigs found Glutalyte® palatable, consuming the majority of the liquid offered.

Physiological normality and translational relevance were attained in several ways. BG remained within normal limits in 10/11 pigs, minimising requirements for glucose supplementation and adverse effects of hypo- or hyperglycaemia on GI motility.^10^ GI motility was deemed normal/acceptable by investigators throughout the study. The GI lumen was empty, expediting stomata surgery, allowing the USCE prototype an unobscured examination field and replicating conditions expected in humans.

Limitations included the lack of a control group, no measurement of Glutalyte® intake/pig and no specific assessment undertaken regarding behavioural changes potentially associated with an impoverished environment. Only female pigs were used according to the demands of the primary study.

Providing a high-carbohydrate liquid diet to pigs as the sole energy source for 36–48 hours before GI surgery and USCE produced a clean, motile small intestine which was suitable for the experiment performed. With appropriate environmental adaptation, pigs demonstrated neither adverse behaviours nor physiological abnormalities. Therefore, this proved a successful way to prepare laboratory pigs for GI surgery and capsule endoscopy studies whilst avoiding aversive procedures, that is, purgative administration and food withdrawal.
